# DECIDE, DISCOVER, AND DO: AN INTERVENTION FOR COMMUNITY-DWELLING INDIVIDUALS WITH DEMENTIA

**DOI:** 10.1093/geroni/igz038.1730

**Published:** 2019-11-08

**Authors:** Gregg Gorzelle, Michael Skrajner, John Zeisel, Cassie Best, Drew Walker

**Affiliations:** 1 Hearthstone Alzheimer Care, Woburn, Massachusetts, United States; 2 Hearthstone Alzheimer Care, Mass, United States; 3 Hearthstone Alzheimer Care, MA, United States

## Abstract

Decide, Discover, and Do!TM (D3) is a mobile application being developed and evaluated in an NIA-funded Phase 1 SBIR project. The goal of D3 is to enhance the quality of life and care for community-dwelling individuals with dementia (IWD) whose primary care partners (CPs) are their family members. D3 consists of (1) video-based, interactive training for family CPs on best practices in dementia care and (2) evidence-based activities for CPs to facilitate with their loved ones. The study examines the impact of D3 training modules on knowledge transfer and the effects of D3 activities on engagement/affect. The activities are unique in that they create an overarching narrative for daily activities that creates a consistent routine, thereby capitalizing on procedural memory. The activities build upon one another, starting with the IWD choosing a topic (e.g., nature) early in the day, followed by the dyad engaging in a tablet-based activity related to the topic (e.g., reading an article about rainforests), and culminating in an experiential activity (e.g., tasting various foods found in rainforests ). In pilot work, when training was implemented with homecare workers, there was a significant increase in knowledge from pre- to post-test (+21.0, p<.01). In addition, similar activities were shown to be highly engaging, with IWD exhibiting significantly higher levels of constructive engagement (+0.40, p<.01), and significant lower levels of other engagement (-0.13, p<.05), when compared to standard programming. This Phase 1 study will be complete by the conference; outcome data will be presented.

